# Causes of death after first time venous thromboembolism

**DOI:** 10.1186/s12959-024-00586-8

**Published:** 2024-02-01

**Authors:** Frida Lonnberg, Andreas Roos, Maria Farm, André Heurlin, Mantas Okas, Bruna Gigante, Anwar J Siddiqui

**Affiliations:** 1 Department of Medicine, Karolinska Institute, Solna, Sweden; 2 Acute and Reparative Medicine Theme, Karolinska University Hospital, Stockholm, Sweden; 3 Karolinska University, Solna, Sweden; 4 Karolinska University Hospital, Solna, Sweden; 5 Acute Medicine, Capio. St. Görans Hospital, Stockholm, Sweden; 6 Department of Cardiology, Danderyd Hospital, Stockholm, Sweden

**Keywords:** Venous thromboembolism, Pulmonary embolism, Deep vein thrombosis, Cardiovascular death, Emergency department visit

## Abstract

**Background:**

Causes of death after first time community-acquired venous thromboembolism (VTE) diagnosed in unselected patients at the emergency department (ED) was investigated.

**Materials and methods:**

The study consists of all patients > 18 years of age who had a visit for any medical reason to any of 5 different ED in Stockholm County, Sweden from 1st January 2016 to 31st December 2017. We have identified all patients with a first registered incident VTE; deep vein thrombosis (DVT) and/or pulmonary embolism (PE) during the study period. Cox regression models were used to estimate hazards ratios (HR) with 95% confidence intervals (CIs) for all-cause mortality and cause-specific death in patients with DVT or PE using all other patients as the reference group.

**Results:**

In total, 359,884 patients had an ED visit during the study period of whom about 2.1% were diagnosed with VTE (DVT = 4,384, PE = 3,212). The patients with VTE were older compared to the control group. During a mean follow up of 2.1 years, 1567 (21%) and 23,741(6.7%) patients died within the VTE and reference group, respectively. The adjusted risk of all-cause mortality was nearly double in patients with DVT (HR 1.7; 95% CI, 1.5–1.8) and more than 3-fold in patients with PE (HR 3.4; 95% CI, 3.1–3.6). While the risk of cancer related death was nearly 3-fold in patient with DVT (HR 2.7; 95% CI, 2.4–3.1), and 5-fold in PE (HR 5.4; 95% CI, 4.9-6.0 respectively). The diagnosis of PE during the ED visit was associated with a significantly higher risk of cardiovascular death (HR *2.2*; 95% CI, 1.9–2.6).

**Conclusion:**

Patients with VTE have an elevated risk of all-cause mortality, including cardiovascular death.

**Supplementary Information:**

The online version contains supplementary material available at 10.1186/s12959-024-00586-8.

## Introduction

Acute venous thromboembolism (VTE) is comprised of deep vein thrombosis (DVT) and pulmonary embolism (PE). VTE is considered the third leading cause of vascular disease after myocardial infarction (MI) and stroke [1]. VTE is seen in all age categories but more frequent among elderly and the incidence of VTE is expected to be rising with an ageing population [2, 3]. Guidelines recommend direct oral anticoagulation (DOAC) over vitamin K antagonist (VKA) in VTE management [4, 5]. DOAC treatment require less dose adjustment, monitoring and dietary restriction, DOAC is also associated with a lower risk of major bleeding events [5].

Mortality and causes of death in patients diagnosed with VTE at the ED are largely unknown in the contemporary era of DOAC. Prior recent studies investigating cause-specific death in VTE-patients have generally been small and have mainly reported short-term mortality. A large study of patients diagnosed with first time VTE between 1980 and 2011 reported a mortality rate ratio within 1 to 10 years of VTE-diagnosis to be 36% higher for DVT and 41% higher for PE compared to the general population [6]. Available data suggest that cancer is the leading causes of death in elderly VTE patients [7]. Deaths in direct conjunction with VTE events are attributed to PE, although there is a huge uncertainty and presumably a high rate of misdiagnosis of the causes of death associated with PE [8]. A prior meta-analysis showed no effect of VTE prevention on mortality, suggesting that VTE can be seen rather as a marker of mortality than the actual cause of death [9].

Clinical course and outcomes vary in VTE-patients depending on underlying diseases and provoking factors [7, 10]. Previous reports indicate that patients with VTE and weak risk factors for VTE such as infection have a higher rate of VTE-recurrence compared to patients with VTE and a strong risk factor such as certain surgery [11]. In patients with VTE and no identifiable risk factor for VTE indefinite anticoagulation should be considered according to guidelines [4]. In Sweden these patients and patients with complicated VTE are usually followed by anticoagulation clinics while most cases of uncomplicated VTE are followed by general practitioners’ centers.

Despite improvement in VTE diagnostics and new treatment options, mortality associated with VTE is still high and remains a major health concern [1, 12]. Recent available data principally relies on short-term follow-up in VTE-patients treated with DOAC [13, 14]. We aimed to investigate all-cause mortality and causes of death among patients with DVT and PE in a large cohort of patients visiting EDs in Stockholm County.

## Materials and methods

### Study population

All patients > 18 years old visiting any of 5 different EDs in Stockholm, Sweden (at Karolinska University Hospital, Danderyd Hospital, South Hospital, S:t Göran Hospital and Södertälje Hospital) for any medical reason from 1st January 2016 to 31st December 2017 were included. Within this cohort, patients with a first-time DVT or PE diagnosis during the study period were identified. Patients were followed until 31st of December 2018. The study was approved by the local Ethics Committee in Region Stockholm and conformed to the ethical guidelines of the 1975 Declaration of Helsinki.

### Data sources

Patients’ data from the ED visit was collected from the central IT department of Region Stockholm after approval from local hospital´s authorities. The patients’ national identity numbers were compiled and sent to the Swedish National Board of Health and Welfare for linkage to data on patients’ comorbidities from the Swedish National Patient Register (NPR: which comprises all health care data of inpatient and outpatient specialist care), prescribed medications from the Prescribed Drug Register and to causes and dates of death from the Cause-of-Death Register. In the Cause-of-Death Register, all causes of death are classified and coded according to the 10th version of the International Classification of Disease (ICD-10).

### Exposure

The exposure was defined as VTE diagnosed in the absence of a priorly registered VTE diagnosis in the NPR during the study period. Patients with VTE were then further categorized into DVT and PE respectively. Patients with concurrent DVT and PE were included in the PE-group. The follow-up for the exposed group started at the first visit with a VTE diagnosis registered and for the unexposed group at the index date i.e., at the time of the first ED visit during the study period.

### Definitions

VTE was defined according to the ICD-codes registered in the NPR during the study period: PE (I26.9 and I26.0) and DVT (I80.2; I80 (I80.0 excluded), I82 (I82.1 excluded). Medications dispensed within 6 months prior to the index date were regarded as baseline medications except DOAC, low-molecular weight heparin (LMWH) and warfarin. All the anticoagulants’ prescriptions claimed within 60 days from the index date were further used to validate ICD codes for VTE. Apixaban, Dabigatran, Rivaroxaban and Edoxaban were the available DOACs for VTE treatment in Sweden during the study period. Comorbidities (diabetes, hypertension, heart failure, chronic obstructive pulmonary disease, chronic kidney disease, stroke, myocardial infarction, atrial fibrillation, and cancer) were defined according to ICD-codes in the NPR with at least one registration as a main or secondary diagnosis in NPR for up to 3 years prior to the index date.

### Outcomes

The primary and secondary outcomes were all-cause mortality and cause-specific mortality, respectively. Mortality was ascertained by using the Causes of Death Register. Cardiovascular death was defined as death with an underlying cause of death in the I-chapter, or R960-R961, according to ICD-10. Cancer-related death was defined as ICD code (C00-C97) in the Causes of Death Register. Any other death was defined as non-cardiovascular non-cancer related death (Supplementary document).

### Statistical analysis

Descriptive statistics were performed using means and standard deviations for summarizing numerical variables, and frequencies and percentages for categorical variables. Incidence rates were calculated for all-cause mortality and cause-specific deaths. Kaplan-Meier curves were constructed to visualize survival from all causes of death and cardiovascular death in the exposed and unexposed groups. The log rank test was used to test for differences between DVT and PE. Cox proportional hazard models were used to estimate the risk of all cause death, cardiovascular death, and cancer related death in the DVT and PE group in reference to the control group. Adjustments were made for age, sex, comorbidities (diabetes, hypertension, heart failure, chronic obstructive pulmonary disease, chronic kidney disease, stroke, atrial fibrillation, cardiovascular disease and cancer) and medications at baseline (anticoagulants, antihypertensive agents, and lipid lowering agents) in the constructed models. P-values < 0.05 were considered significant in the analysis. R version 4.3.1 and SAS version 9.4 TS Level 1M8 were used for all statistical analysis and graphics.

## Results

### Study population

**Fig. 1:**
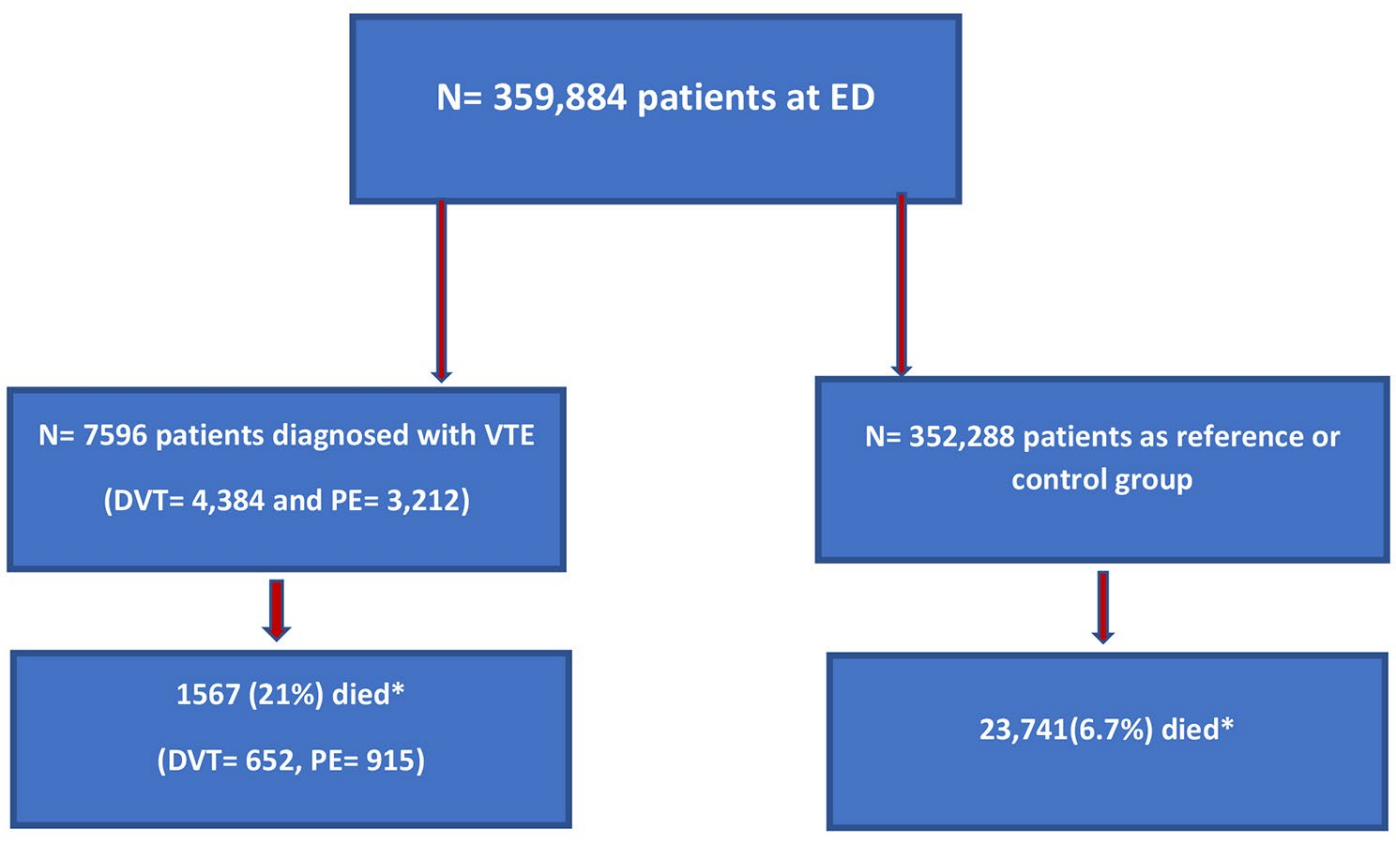
Patients selection flowchart from the entire population cohort

During the study period altogether 359,884 patients visited EDs in Stockholm of whom 7596 (2.1%) were diagnosed with a first time VTE (Fig. 1). The patients with VTE were older compared to the control group. The most common comorbidities among the VTE-patients were hypertension, diabetes, atrial fibrillation, and cancer. DOAC was the most frequent anticoagulation prescribed followed by LMWH and Warfarin. At index date 24,371(6.9%) of the patients in the control group had a cancer diagnosis whereas 797 patients (18.2%) in the DVT group and 853 patients (26.6%) in the PE group had a cancer diagnosis (Table 1).

**Table 1:** Baseline characteristics of the patients included in the study

Variables	All patients	Control	DVT	PE
Number of patients, n (%)	359,884 (100)	352,288 (97.9)	4,384 (1.2)	3,212 (0.9)
Age, years (mean, SD)	48.5 (12.2)	48.4 (12.1)	53 (13.3)	55.2 (13.8)
Age 18–49	174,878 (48.6)	173,365 (49.2)	1,033 (23.6)	480 (17.6)
Age 50–79	144,238 (40.1)	139,663(39.6)	2,564(58.5)	2,011 (62.6)
Age 80+	40,786 (11.3)	39,260 (11.1)	787 (18.0)	721 (22.4)
Male sex, n (%)	157,102 (43.7)	153, 321(43.5)	2,182 (49.8)	1,599(49.8)
Cardiovascular disease	15,053(4.2)	14,556(4.1)	228(5.2)	269(8.4)
Atrial_fibrillation	18,654 (5.2)	18,181 (5.2)	256 (5.8)	217 (6.8)
COPD	8,023 (2.2)	7,583 (2.2)	175(4.0)	265 (8.3)
Diabetes	20,912(5.8)	20,258 (5.8)	333(7.6)	321 (10.0)
HF	10,916 (3.0)	10,471(3.0)	190(4.3)	255(7.9)
Hypertension	43,977(12.2)	42,158(12.0)	144 (3.0)	175 (5.6)
Obesity	7564 (2.1)	1,572 (0.4)	15 (0.3)	10 (0.3)
Renal_disease	7115 (2.0)	6762 (1.9)	199 (4.5)	154(4.8)
Peripheral_Vascular_disease	3093(0.9)	2969 (0.8)	64 (0.4)	60 (0.3)
Liver disease	2416 (0.7)	2308 (0.7)	76 (1.7)	32(1.0)
Cancer	26,021(7.9)	24,371(6.9)	797(18.2)	853(26.6)
Stroke/ischemic	9890 (2.7)	9456 (2.7)	48 (1.0)	26 (0.82)
Anithypertensives	107,015(29.7)	103,544(29.4)	1811(41.3)	1660(51.7)
Insulin	12,133(3.4)	11,804(3.4)	165(3.8)	164(5.1)
Oral diabetes agent	17,269(4.8)	16,863(4.8)	207(4.7)	199(6.2)
Lipid_lowering	46,600 (12.9)	45,310(12.9)	683(15.6)	607(18.9)
Aspirin	35,168(9.8)	34,086(9.7)	555 (12.7)	527(16.4)
LMWH	17,547(4.9)	13,356(3.8)	2728(62.2)	1463(45.5)
DOAC	12,609(3.5)	9,293(2.6)	1744(39.8)	1572(48.9)
Warfarin	7,728(2.1)	7,202(2.0)	210(4.8)	316(9.8)

MI = myocardial infarction, CIHD = Chronic ischemic heart disease, COPD = chronic obstructive pulmonary disease, HF = heart failure, CVD = cardiovascular disease, LMWH = low molecular weight heparin, DOAC = direct oral anticoagulation, DVT = deep vein thrombosis, PE = pulmonary embolism

LMWH, DOAC and Warfarin: baseline plus 60 days from index date

### All-cause mortality

**Fig. 2:**
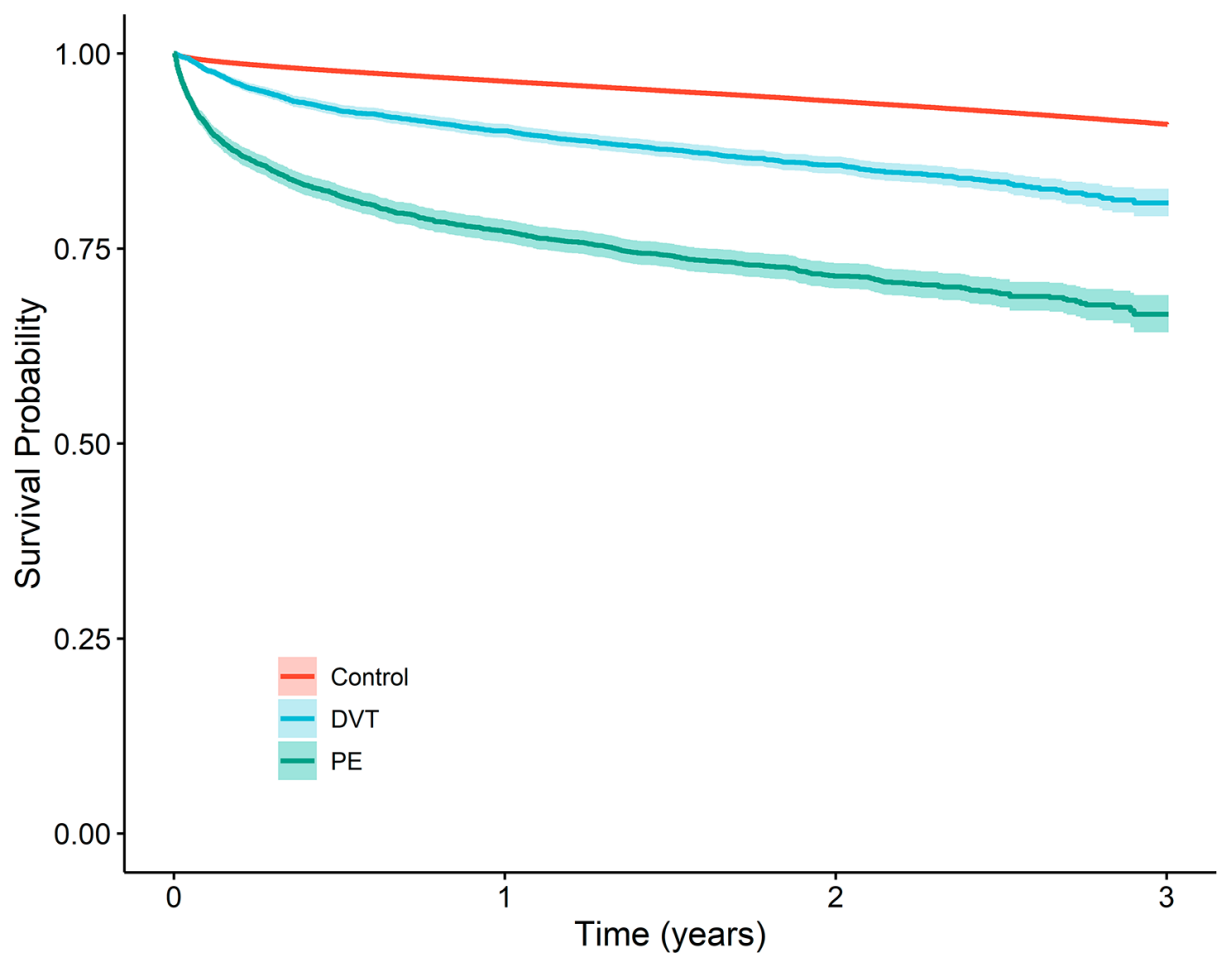
Kapplan-Meier graph showing overall mortality

During a mean follow-up of 2.1 years, more than one-fourth (28.5%) of PE patients died, while 14.9% and 6.7% of patients were died in the DVT group and in the control group, respectively (Fig. 2, Table 2a). The all-cause mortality rate was more than doubled in the DVT and PE groups in comparison to the control group (Table 2b). Women with DVT and PE might experience higher overall and cardiovascular death compared to men with DVT and PE. However, when age is considered, the mortality rate become comparable between both genders (data not shown). The risk of all-cause mortality was higher for both DVT, and PE patients compared to the control group after adjusting for age and sex, and in the fully adjusted model (Table 3).

**Table 2a:** Cumulative deaths, non-cardiovascular, cardiovascular, all cancer and other deaths reported among the 359,884 patients who sought care at a participating emergency department in Stockholm, Sweden from 1st January 2016 to 31th December 2017. In parenthesis is the proportion of deaths in relation to the number of patients in each group: control, DVT, PE and total population respectively

	Control (*n* = 352,288)	DVT (*n* = 4,384)		PE (*n* = 3,212)			Total (*n* = 359,884)		
**All-cause mortality**	23,741(6.7)	652 (14.9)		915 (28.5)			25,307 (7.0)		
**Non-CV death**	16,599 (4.7)	535 (12.2)		735 (22.9)			17,868 (5.0)		
**CV death**	7,142(2.0)	117 (2.7)		180 (5.6)			7,439 (2.1)		
**All cancer death**	6,695 (1.9)	348 (7.9)		492 (15.3)			7,535 (2.1)		
**Other death**	9,904 (2.8)	187 (4.3)		243 (7.6)			10,333 (2.9)		

**Table 2b:** Number (%) of deaths and incidence rates (95% CI) per 100 person-years stratified by outcome (i.e., control, DVT, and PE) and type of death (i.e., all-cause, non-CV death, CV death, all cancer death, and other death) among 359,884 patients who sought care at a participating emergency department in Stockholm, Sweden during 1st January 2016 to 31st December 2017

Type of death	n (%)	Rate/100 py (95% CI)		
	Total	Control	DVT	PE
All cause death	25,307 (7.0)	3.2 (3.1–3.2)	8.0 (7.4–8.6)	17.9 (16.8–19.1)
Non-CV death	17,868 (5.0)	2.2 (2.2–2.3)	6.5 (6.0–7.1)	14.4 (13.4–15.5)
CV death	7,439 (2.1)	1.0 (0.9-1.0)	1.4 (1.2–1.7)	3.5 (3.0–4.1)
All cancer death	7,535 (2.1)	0.9 (0.9–0.9)	4.2 (3.8–4.7)	9.6 (8.8–10.5)
Other death	10,333 (2.9)	1.3 (1.3–1.4)	2.3 (2.0–2.6)	4.8 (4.2–5.4)

CI = confidence interval, CV = cardiovascular, VTE = venous thromboembolism, PE = pulmonary embolism

**Table 3:** Long term risk of death due to CV and cancer in DVT and PE patients in the study. Multivariable model adjusted by comorbidities (diabetes, hypertension, heart failure, chronic obstructive pulmonary disease, chronic kidney disease, stroke, atrial fibrillation, and cancer) and medications at baseline (anticoagulants, antihypertensive, lipid lowering). CI = confidence interval, CV = cardiovascular, DVT = deep vein thrombosis, PE = pulmonary embolism, HR = hazard ratio

All-cause mortality	Control	DVT	PE
Crude HR (95% CI)	Ref	2.5(2.3–2.6)	5.5 (5.1–5.8)
Age- and sex-adjusted HR (95% CI)	Ref	1.6(1.5–1.8)	3.0(2.9–3.3)
*Multivariable adjusted HR (95% CI)		1.7 (1.5–1.8)	3.4 (3.1–3.6)
**CV death**			
Crude HR (95% CI)	Ref	1.5 (1.2–1.8)	3.5 (3.0-4.1)
Age- and sex-adjusted HR (95% CI)	Ref	0.9 (0.8–1.1)	2.0(1.7–2.2)
*Multivariable adjusted HR (95% CI)		1.0 (0.9–1.3)	2.2(1.9–2.6)
**All cancer death**			
Crude HR (95% CI)	Ref	4.6 (4.2–5.1)	10.4 (9.5–11.4)
Age- and sex-adjusted HR (95% CI)	Ref	3.1 (2.8–3.4)	6.0 (5.5–6.6)
*Multivariable adjusted HR (95% CI)		2.7 (2.4–3.1)	5.4 (4.9-6.0)

### Cancer-related death and cardiovascular death

**Fig. 3:**
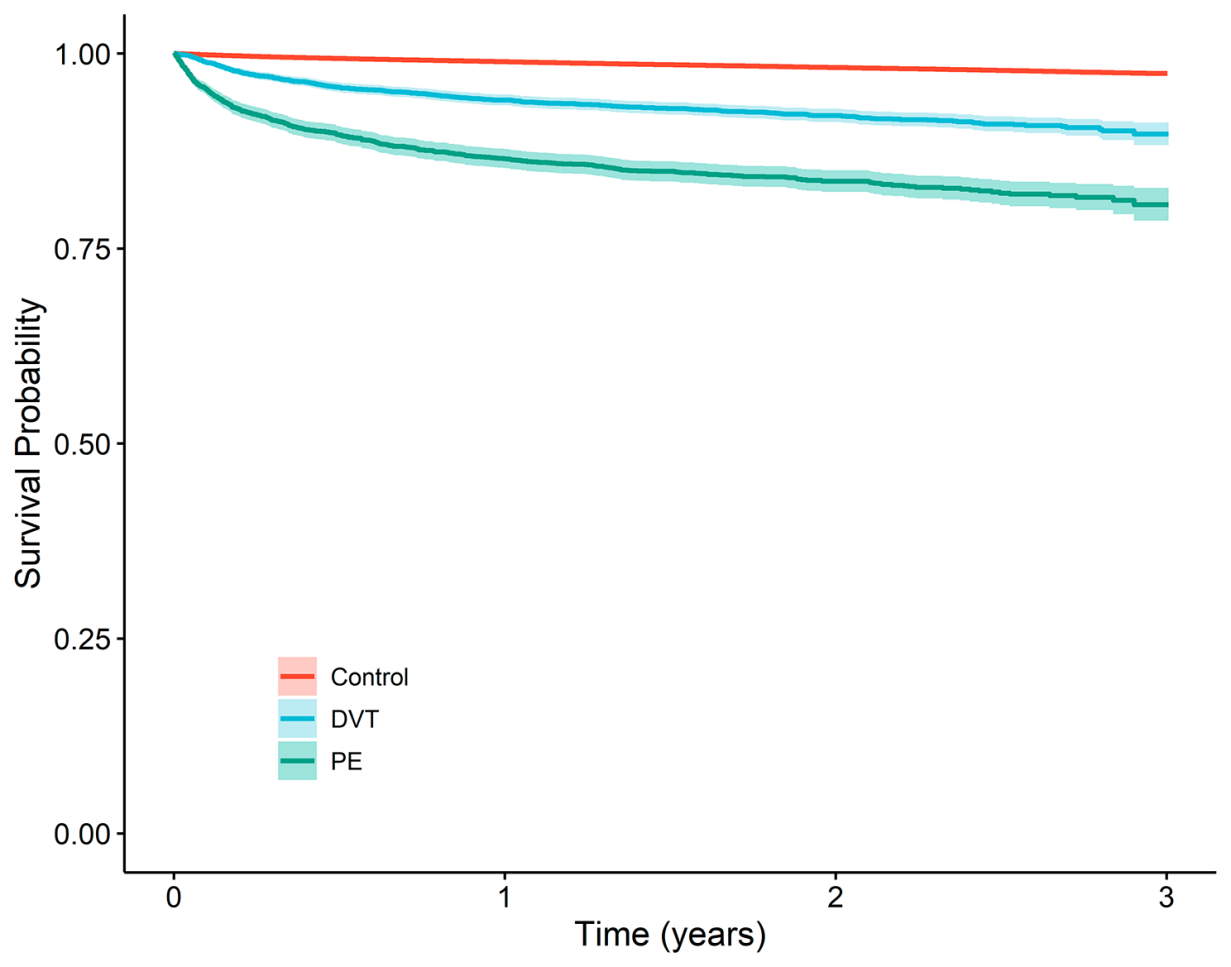
Kapplan-Meier graph showing cancer related mortality

In the control group, 6695 (1.9%) patients died of cancer-related causes within the study period compared to 348 (7.9%) and 492 (15.3%) patients in the DVT and PE group, respectively (Fig. 3, Table 2a). The fully adjusted risk of cancer-related death was 4-fold higher in the DVT group and almost 10-fold higher in the PE group compared to the control group (HR 4.2, 95% CI: 3.8–4.7; and HR 9.6, 95% CI: 8.8–10.5) respectively (Table 3). The rate of non-cancer related death was higher in both the DVT, and PE group compared to the control group (Table 2b).

**Fig. 4:**
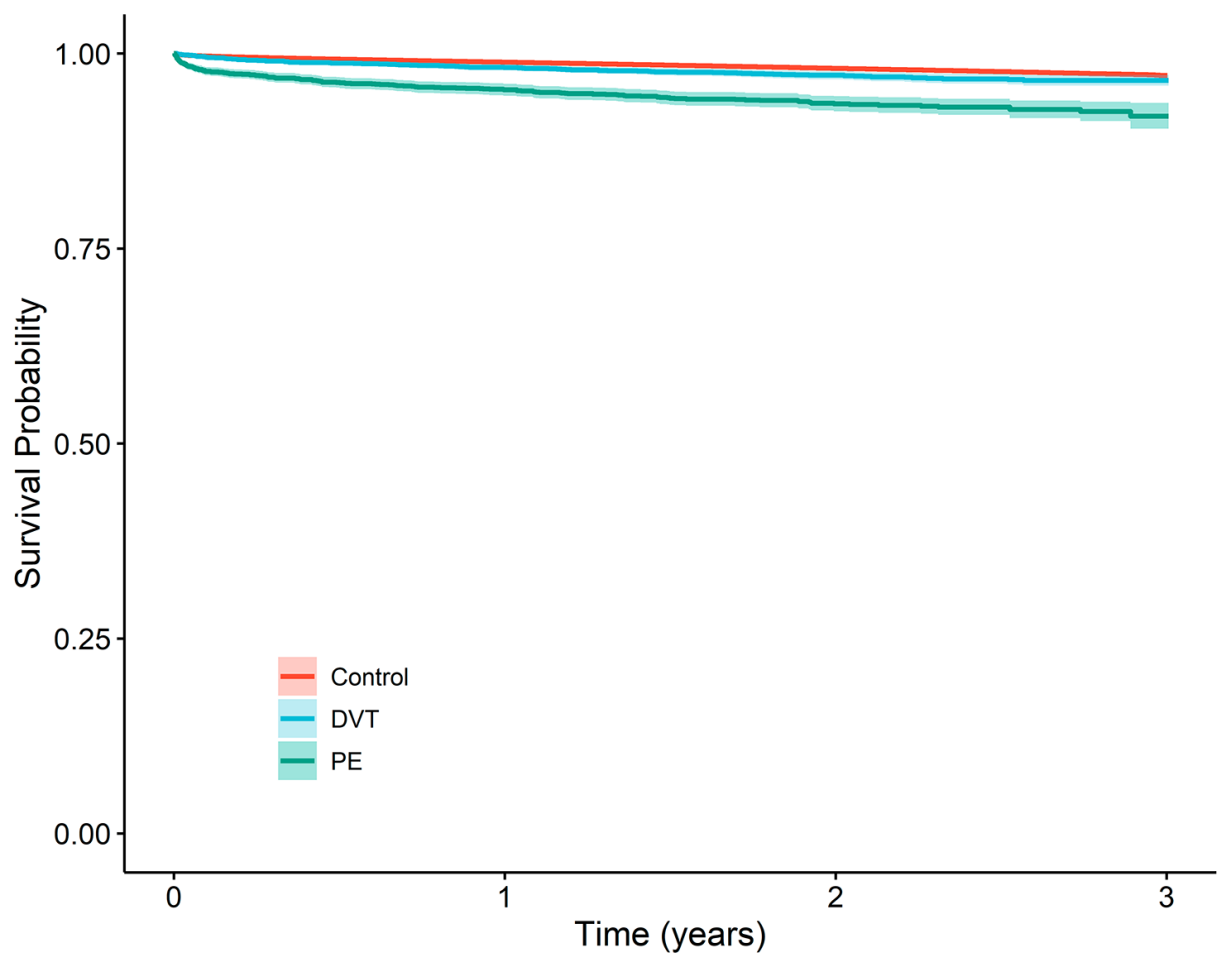
Kapplan-Meier graph showing cardiovascular related mortality

A total of 7142 (2.0%) patients died of cardiovascular cause in the control group, compared to 117 (2.7%) patients in the DVT group and 180 (5.6%) patients in the PE group (Fig. 4, Table 2a). The cardiovascular mortality rate was higher in the PE group compared to the control group; and apparently no difference was observed between the DVT group and the control (Table 2b). The fully adjusted risk of cardiovascular death was more than 2-fold higher (HR: 2.2, 95% CI: 1.9–2.6) in patients with PE compared to the control group (Table 3). In patients with DVT the fully adjusted risk of cardiovascular death was not increased in comparison to the control group (HR: 1.0, 95% CI: 0, 9 − 1, 3).

Due to a significant age difference (> 7 years) and a higher incidence of cancer among VTE patients, we further analyzed the age-adjusted incidence by stratifying data into 10-year age intervals to examine mortality rates among individuals with DVT and PE. Data revealed that as the age interval increased (starting from 50 to 59 and older groups), there was a corresponding increase in mortality among patients with DVT and PE in terms of all cause death, cardiovascular-related mortality, and death attributed to cancer (Table 4).

**Table 4:** Age adjusted mortality incidence by stratification in 10 years age interval. Age specific mortality per 100 p-years

	Age	All cause death	Non-CV death	CV death	All cancer death	Other death
Control	18–49	0.2 (0.2–0.2)	0.0 (0.0–0.0)	0.2 (0.2–0.2)	0.1 (0.1–0.1)	0.2 (0.2–0.2)
	50–59	0.8 (0.8–0.9)	0.1 (0.1–0.1)	0.8 (0.8–0.9)	0.4 (0.4–0.5)	0.5 (0.5–0.6)
	60–69	2.2 (2.1–2.3)	0.3 (0.3–0.3)	2.1 (2.0–2.1)	1.2 (1.1–1.2)	1.2 (1.1–1.3)
	70–79	4.7 (4.6–4.8)	0.8 (0.8–0.9)	4.2 (4.1–4.3)	2.2 (2.1–2.3)	2.8 (2.6–2.9)
	80+	15.5 (15.3–15.8)	4.3 (4.2–4.5)	11.4 (11.1–11.6)	3.2 (3.1–3.3)	11.3 (11.1–11.6)
DVT	18–49	1.2 (0.8–1.6)	0.1 (0.0-0.2)	1.2 (0.9–1.7)	0.8 (0.6–1.3)	0.4 (0.2–0.9)
	50–59	2.7 (2.1–3.6)	0.1 (0.0-0.3)	2.8 (2.2–3.8)	2.0 (1.5–2.8)	0.9 (0.5–1.7)
	60–69	5.6 (4.8–6.7)	0.3 (0.2–0.5)	5.8 (4.8–6.9)	4.3 (3.6–5.3)	1.6 (1.1–2.4)
	70–79	7.4 (6.5–8.5)	0.6 (0.4–0.9)	7.3 (6.3–8.5)	4.9 (4.1–5.9)	2.9 (2.1–3.8)
	80+	16.1 (14.3–18.0)	3.1 (2.5–3.8)	12.4 (10.8–14.2)	4.9 (4.0–6.1)	10.2 (8.5–12.2)
PE	18–49	2.4 (1.7–3.3)	0.1 (0.0-0.3)	2.4 (1.7–3.4)	1.5 (1.0–2.3)	1.2 (0.6–2.2)
	50–59	6.7 (5.4–8.4)	0.4 (0.2–0.8)	6.7 (5.3–8.5)	4.6 (3.5–6.1)	2.4 (1.5–3.9)
	60–69	9.8 (8.5–11.4)	0.5 (0.3–0.8)	10.0 (8.5–11.6)	7.4 (6.2–8.8)	2.7 (1.8–3.8)
	70–79	14.2 (12.7–15.9)	1.1 (0.8–1.5)	13.5 (12.0–15.3)	8.6 (7.5–9.9)	6.1 (4.9–7.6)
	80+	26.6 (24.1–29.3)	4.2 (3.6–5.0)	20.6 (18.3–23.1)	8.6 (7.2–10.2)	16.7 (14.2–19.6)

## Discussion

In a large cohort of patients in Stockholm County of inhabitant over 2.2 million we found that a final diagnosis of PE was associated with more than 3-fold increased risk of all-cause mortality, and 2.2-fold increased risk of cardiovascular death compared to patients without VTE. Patients with DVT had an increased adjusted risk of all-cause mortality, but no increased risk of cardiovascular death. Cancer-related mortality was increased both in DVT and PE patients.

Most prior studies on mortality and causes of death in patients with VTE are conducted before the introduction of DOAC as a treatment of VTE. In two small cohort studies the 1-year all-cause mortality was 22% and 24% in patients with a first time VTE [15, 16]. A large prospective cohort study published in 2013 showed a 3-month all-cause mortality of 8% in patients with VTE [17]. A metanalysis including 10 trials and 35,029 patients had similar case fatality rate among patients with VTE that were treated with DOACs as we report in our study [18]. In Sweden most of the community-acquired DVT-cases are diagnosed as outpatient facilities (often situated or connected to a hospital ED) and few at general practitioner’s centers, while PE is most often diagnosed at EDs and in hospitalized patients. Considering that our study population is solely comprised of patients presenting to the ED, the severity of disease in patients diagnosed with VTE may differ from that in other study settings.

The observation of a higher risk of cancer-related death in the DVT and PE-group compared to the control group was expected. Our results are consistent with the results from a prior large cohort study in which cancer was the most frequent cause of death in VTE patients within 30 days and at 1 year [10]. In our study the number of cancer-related deaths exceeded that of patients with known cancer disease at baseline within the VTE-group which suggests that a considerable proportion of patients were diagnosed with cancer after the VTE-diagnosis. An earlier study showed that the survival of patients diagnosed with cancer at the time of the VTE-diagnosis is poorer than that of matched control patients with the same type and stage of cancer [19].

We found that the adjusted risk of cardiovascular death was significant higher in patients with PE compared to patients in the control group, whether this is attributed to acute death from PE or from cardiovascular events such as acute heart failure, MI, or stroke, is unknown. The number of PE-related deaths are generally few, and approximately contribute to less than 0.4% of all deaths in Sweden [20]. Due to a steady decline in autopsy rates in Sweden [21] the cause of death in many of the patients with PE is not certain. A prior study found an in-hospital mortality rate of only 1,1% in patients presenting with PE in the ED [22], which might suggest that acute death from PE could not fully explain the increased risk of cardiovascular death among patients with PE observed in our study. A previous small scale study reported that 40% of mortality is related to cardiovascular disease after PE, with a mean follow-up time of 3.8 years [23]. An increased risk of arterial cardiovascular events in patients with unprovoked VTE was previously reported in a meta-analysis [24]. If this can be assigned to similar risk factors for VTE and atherothrombotic disease, or if VTE could act as a trigger for atherothrombotic disease is unknown. Another meta-analysis showed a positive association between the presence of major risk factors of atherothrombotic disease, especially hypertriglyceridemia, and the risk of a VTE event [25]. In our study the increased risk for cardiovascular death remained high after adjustments with the comorbidities known for cardiovascular risk factors.

Age-standardized PE-related mortality has been decreasing in Europe since 2000 [26] and according to the Swedish healthcare annual statistic report a trend in reduced VTE-related mortality has been observed in the last few years [20]. We did not have data before the introduction of DOAC, which limited our ability to investigate a potential change in VTE related mortality associated with current treatment strategies.

We do acknowledge that the observed increased all-cause mortality risk in VTE-patients was reflected in all categories of cause-specific death. A more detailed categorization of CV death would have been more informative; yet the absolute numbers of outcomes were relatively small which limited the ability to explore more details on cause-specific deaths. However, age stratified analysis revealed that as the age interval increased (starting from 50 to 59 and older groups), there was a corresponding increase in mortality among patients with DVT and PE in terms of all cause death, cardiovascular-related mortality, and death attributed to cancer. Nonetheless, we believe that the increased adjusted risk of CV death is a novel finding which need further exploration in VTE patients.

### Strengths and limitations of the study

This is an observational cohort study, cautious interpretations of data in the context of any clinical setting should be considered, mainly because of the inherent risk with residual confounding that we could not control for. The study was conducted in a large cohort of patients, although not so long follow-up time but it allowed us to calculate risk estimates the events with high precision. A strength of our study is that the source of our data, the Swedish registers, are known to be of high quality, including the Swedish Prescribed Drug Register [27–29]. The Cause-of-Death Register has excellent nationwide coverage with virtually no loss to follow-up [21]. There are however reports of some degree of misclassification of VTE diagnoses in the Swedish NPR, specifically for DVT but also to some extent for PE [30]. Taking misclassification into consideration, it affects only 1.9% of DVT cases and 0.3% PE cases, resulting in slightly higher mortality rates that has been reported in our study. However, it is important to point out that the registration of causes of deaths according to ICD codes in the Causes of Death Register is conducted independently from any registrations in the National Patient Register.

We did not have data available from primary healthcare, as this is not included in the Swedish NPR. Consequently, misclassification of the exposure may have occurred, i.e., as some patients may have had a DVT diagnosed solely within primary care during the study period or even before our study started. Although we were able to control for the most important confounders in our statistical models, we do not have information regarding stage or type of cancer. It was not possible to conclude with certainty that the severity of cancers was similar between the DVT and PE group, mortality differences could be related to a worse prognosis of the cancer itself rather than the VTE. We acknowledge that both the differences in age, burden of cardiovascular comorbidities and prevalent cancer at baseline would all be contributing factors to the observed differences in outcomes between the groups. Competing risk events in the analysis of causes of death was not addressed in our analysis, however, we have adjusted for all these variables in our statistical models. In our belief the fully adjusted models sufficiently handle the confounding effect of these factors and provide adequate estimates on the association between exposure and the outcomes of interest. We consider that the findings in our study on Swedish data are generalizable to other countries with similar health care system.

## Conclusion

In this large cohort study with unselected patients in the ED we found an increased adjusted risk of death among patients with VTE. Besides an expected association between a VTE diagnosis and cancer-related death we observed that patients with PE had an elevated adjusted risk of cardiovascular deaths.

### Electronic supplementary material

Below is the link to the electronic supplementary material.

Supplementary Material 1

## Data Availability

Open access to data is not allowed according to the Swedish law. According to the Swedish Ethics Review Act, the General Data Protection Regulation, and the Public Access to Information and Secrecy Act, patient data can only be made available, after legal review, to researchers who meet the criteria for access to this type of confidential data.
