# Supplementary material for: Causes of death after first time venous thromboembolism
Source: Thromb J. 2024 Feb 1;22:16. doi: 10.1186/s12959-024-00586-8 (PMC10832181; doi:10.1186/s12959-024-00586-8)
Supplement: Supplementary file 1 — Supplementary Material 1 [file 12959_2024_586_MOESM1_ESM.docx]

**Supplement document: 1**

Definitions of baseline co-morbidities by ICD-10, primary care codes and procedure codes.

| **Diagnosis** | **ICD-code beginning with** |
| --- | --- |
| Alcohol abuse | E244, F10, G312, G621, G721, I426, K292, K70, K860, O354, P043, Q860, T51, Y90-91, Z502, Z714 |
| Anaemia | D50-64 |
| Any severe bleed | I60-62, I690-I692, S064-S066, I850, I983, K25-28 (sub codes 0-2 and 4-6 only), K625, K922, D500, D629, J942, I312, H431, H356 |
| Atrial fibrillation | I48 |
| Cancer | entire C-series |
| COPD/Emphysema | J43-44 |
| Dementia | F00-F03 |
| Diabetes | E10-E14 |
| Frequent falls (more than one registration) | W00-19 |
| Gastric duodenal bleeding | K25-28 (sub codes 0-2 and 4-6 only) |
| Heart failure | I50 |
| Hypertension | I10-I15 |
| Ischemic stroke, arterial embolism, and stroke, unspecified | I63, I64, I679, I693, I694, I698, I67-, I69-, Z866A, Z866B, Z867C, G450, G451, G452, G453, G458, G45.9, G45-, I74 |
| Intracranial bleeding | I60-I62, I690-I692, S064-S066 |
| Liver disease | K70-77 |
| Obesity | E65-66 |
| Renal disease | N17, N183, N184, N185, N189 |
| Vascular disease/MI and PVD | I20-I25, I70, I739 |
| Venous thromboembolism | I26, I80 (I80.0 excluded), I82 (I82.1 excluded), I27.82 |
| Hyperlipidemia | E78.5, E78.0, E78.1,E78.2, E78.2X, E78.8, E78.9,E78.4, |

**ATC-codes of the studied treatments**

| **Treatment** | | **ATC-code beginning with** | | | |
| --- | --- | --- | --- | --- | --- |
| Acetylsalicylic acid (aspirin) | | B01AC06 | | | |
| Antidepressants | | N06A | | | |
| Antihypertensive treatments | | C03 C07 C08 C09 | | | |
| Anticonception | | G03A G03F | | | |
| Anticonception2 | | G03AA G03AB | | | |
| Clopidogrel | | B01AC04 | | | |
| Lipid lowering treatments | | C10 | | | |
| Low molecular weight heparin (LMWH) | | B01AB04 B01AB05 B01AB10 | | | |
| Insulin | | A10A | | | |
| Direct oral anticoagulant (DOAC) | | B01AE07 B01AF01 B01AF02 B01AF03 | | | |
| Actilyse | | B01AD02 | | | |
| Oral diabetes treatments | | A10B | | | |
| Prasugrel | | B01AC22 | | | |
| Ticagrelor | | B01AC24 | | | |
| Protonpump inhibitors | | A02BC | | | |
| Warfarin | | B01AA | | | |
| ICD-10 code | Cancer type | |  |  |  |
| C44 | Skin cancer | |  |  |  |
| C61 | Prostate cancer | |  |  |  |
| C50 | Breast cancer | |  |  |  |
| C77 | Metastatic lymph node | |  |  |  |
| C78 | Metastasis in lung, thorax, liver or  other gastrointestinal organs | |  |  |  |
| C79 | Other sites of metastasis | |  |  |  |
| C34 | Lung cancer | |  |  |  |
| C18 | Colon cancer | |  |  |  |
| C67 | Bladder cancer | |  |  |  |
| C20 | Rectal cancer | |  |  |  |
